# A Breath of Fresh Air: A New Analysis of an Age-Old Question

**DOI:** 10.1016/j.chpulm.2025.100150

**Published:** 2025-02-24

**Authors:** Berty Baskaran, Ryan Butzko

**Affiliations:** Department of Medicine, Division of Pulmonary, Critical Care and Sleep Medicine, SUNY Upstate Medical Center, Syracuse, NY

COPD-OSA overlap syndrome is a relatively common comorbid respiratory disorder, only becoming more common with rising levels of obesity. This places an increased financial burden on the health care system due to increased rates of hospitalization and mortality.^1^ Despite the fact that we have an effective therapy for OSA, current data are decidedly lacking in demonstrating a clinical benefit regarding positive airway pressure (PAP) therapy. Prior randomized controlled trials^2^, ^3^, ^4^ have failed to show a long-term benefit regarding PAP therapy. Post hoc analyses have demonstrated some benefit regarding cardiovascular risk in patients who are adherent to therapy^2^; however, definitive positive data remain elusive. Some papers (eg, the Adaptive Servo-Ventilation for Central Sleep Apnea in Systolic Heart Failure trial)^5^ have even demonstrated harm to patients. Several critiques have been mentioned to explain the lack of benefit in these trials (eg, poor patient adherence to therapy, excluding those patients most likely to benefit). As such, the sleep medicine community has been challenged to create innovative strategies to assess the potential benefits to patients using PAP therapy.

In the March issue of *CHEST Pulmonary*, Tellez et al^6^ strive to determine whether home noninvasive ventilation (NIV) impacts the rate of severe exacerbations of COPD. This study design is becoming increasingly popular as a real-world analysis, which is well suited for the OSA population given the low adherence rates typical of this population and the ethical issues that have arisen with the aforementioned randomized controlled trials.

This paper looks at bilevel pressure ventilation and its effect on reducing hospitalizations in overlap syndrome. The choice to only study bilevel pressure ventilation was surprising. On the surface, one would expect that patients with COPD-OSA overlap syndrome would benefit significantly from bilevel pressure ventilation because this mode provides increased ventilatory support. However, CPAP could potentially provide the same benefit, by the simple fact that increased airway pressure would allow for further emptying of the lungs by preventing early airway collapse while simultaneously treating obstructive apneas. This approach is also more cost-effective. A meta-analysis study done by Czerwaty et al^7^ looked at 38 studies that included a diagnosis of OSA based on polysomnography data and COPD diagnosis based on spirometric values from Global Initiative for Chronic Obstructive Lung Disease guidelines to evaluate the effect of this overlap syndrome. The prevalence in development of hypertension, coronary artery disease, and other cardiovascular disease appeared to be greater in patients with overlap syndrome as opposed to either disease individually. The truly interesting feature of this meta-analysis arises from the section where they evaluate the effects of CPAP in overlap syndrome. The patients with overlap syndrome who were treated with CPAP had lower rates of mortality and decreased hospitalization due to COPD exacerbations. One year of CPAP usage was found to have improvement in Pao_2_ and Paco_2_, including in patients with hypercapnia at baseline.

A study by Sterling et al^8^ also looked at the COPD and OSA overlap group and found that there was reduction in hospitalization, emergency department visits, exacerbations, and health care costs in those who were adherent to CPAP. This, in conjunction with the meta-analysis previously mentioned, points to the importance of studying the comparisons. Further trials are needed to identify the pertinent risk factors for rehospitalization and to compare CPAP with bilevel pressure ventilation in this population. In cases of patients with obesity hypoventilation syndrome with severe OSA, who by definition have chronic hypercapnic respiratory failure, the criterion standard is to manage with CPAP initially and advance to bilevel pressure ventilation devices if CPAP is ineffective at controlling the gas exchange abnormalities. Comparing the cost-effectiveness of each therapy would favor CPAP as initial therapy for this syndrome.

In this paper, over one-half of the roughly 24,000 patients with COPD-OSA overlap carried an additional diagnosis of morbid obesity (53.75%), which greatly increases the risk of obesity hypoventilation syndrome. Therefore, it invites the question of whether these patients had hypercapnia at baseline, and whether that is the driving force behind the benefit. Because no blood gas analyses could be obtained with this study design, we will continue to wonder.

The importance of this paper is in detailing the increasing prevalence of COPD-OSA overlap syndromes and the need for adequate management. Fortis et al^9^ performed a retrospective study where they mention the underutilizations of home NIV for patients who present with hypercapnic respiratory failure secondary to COPD. They noted that approximately 50% of patients with at least 1 COPD-related hospitalization have compensated hypercapnic respiratory failure, but only 8% of these patients actually end up being treated with home NIV. This speaks on the importance of evaluating for hypercapnic respiratory failure while the individual is an inpatient and assessing for risk factors of underlying OSA because there is a high degree of overlap in the patient population. This can then be transitioned into appropriate ordering of NIV in these patients before discharge and having adequate follow-up to make sure sleep testing gets done, either as an inpatient or outpatient.

Overall, the current paper does demonstrate a clear benefit regarding COPD exacerbations and hospitalizations with the utilization of bilevel pressure ventilation therapy, a positive impact that is sorely needed in the literature. The reason behind the benefit needs to be further elucidated, and hopefully this paper serves as a hypothesis-generating catalyst. Whether the same impact may be felt with other modalities of PAP therapy remains to be seen.

## Financial/Nonfinancial Disclosures

None declared.
